# Online class or flipped-jigsaw learning? Which one promotes academic motivation during the COVID-19 pandemic?

**DOI:** 10.1186/s12909-021-02929-9

**Published:** 2021-09-21

**Authors:** Alireza Mortezaei Haftador, Fatemeh Shirazi, Zinat Mohebbi

**Affiliations:** 1 Student Research Committee, School of Nursing and Midwifery, Shiraz University of medical sciences, Shiraz, Iran; 2 Community Based Psychiatric Care Research Center, Department of Nursing, School of Nursing and Midwifery, Shiraz University of Medical Sciences, Shiraz, Iran; 3 Department of Nursing, School of Nursing and Midwifery, Shiraz University of Medical Sciences, Shiraz, Iran

**Keywords:** COVID-19, Motivation, Online education, Flipped classroom, Jigsaw, Students

## Abstract

**Background:**

Due to the progress in COVID-19, education has undergone a huge change all around the world, leading all universities to move towards distance learning. In this context, the majority of instructors tend to make use of the educational methods that maintain and improve students’ motivation and, consequently, promote their academic performance. This study aimed to compare the effects of synchronous online class and the combination of flipped and jigsaw methods on students’ academic motivation.

**Methods:**

This quasi-experimental study was conducted on 84 BSc nursing students who had entered Shiraz University of Medical Sciences in two different years. One group was educated in a synchronous online class, while the other group was educated using a combination of flipped and jigsaw methods. Both classes were enrolled in online classes due to the COVID-19 pandemic. The participants’ academic motivation was evaluated using Harter’s Academic Motivation Scale.

**Results:**

The results revealed no significant difference in the intrinsic and extrinsic dimensions of academic motivation in the synchronous online class group before and after the intervention. However, a significant increase was observed in the mean scores of academic motivation (*p* = 0.002) and its intrinsic (*p* = 0.003) and extrinsic (*p* = 0.031) dimensions in the flipped-jigsaw method group after the intervention. Moreover, the mean scores of academic motivation (*p* = 0.007) and its intrinsic (*p* = 0.038) and extrinsic (*p* = 0.010) dimensions were significantly higher in the flipped-jigsaw method group compared to the synchronous online class group after the intervention.

**Conclusions:**

Since the COVID-19 pandemic has led educational institutions to use virtual education methods, the combination of flipped and jigsaw methods may improve students’ academic motivation in distance learning.

## Background

COVID-19 is a viral disease, against which no one is safe [1, 2]. In order to control the prevalence of this disease, governments had to close educational institutions temporarily, which affected 91% of the student population around the world [3]. In other words, this disease resulted in a huge change in teaching and learning [2]. Nevertheless, one of the main stimulants that maintain students’ stability and effort in the learning process and ultimately contribute to their success in the education process is motivation [4]. Experts have divided motivation into two main groups, namely intrinsic and extrinsic motivation, which are related to learning and academic motivation [5]. In intrinsic motivation, individuals show some behaviors in order to achieve the sense of competence and determine their faith. Behaviors derived from intrinsic motivation take place in such circumstances as overcoming challenges and reducing incompatibilities or on the basis of environmental conditions [6]. Extrinsic motivation is created due to external incentives and rewards [7]. For example, the existence of specified rules and regulations may result in behaviors originating from extrinsic motivation [6]. Psychologists have stated that motivation should be taken into consideration in education due to its relationship with learning. They have also presented academic motivation as one of the primary structures for defining this type of motivation [5]. In fact, motivation acts as a stimulus for learners for completion of a task, attaining an aim, and achieving a high degree of competence in their professions [8]. Great attention has been paid to academic motivation in the studies carried out on teaching and learning around the globe [9]. Evidence has indicated that higher motivation of students resulted in their greater attempts in difficult affairs as well as their resistance in the face of obstacles, which improved their skills and promoted their scientific performance [10]. Furthermore, academic motivation was associated with educational progress among medical students and was effective in their educational achievements [11]. Overall, the required energy for carrying out the academic tasks was produced by motivation [8].

Teaching methodology is one of the strategies for creation of motivation and promotion of learning. Since instructors play the key role in the teaching-learning process, they need to have sufficient knowledge about new teaching methods in order to be able to teach appropriately on the basis of learners’ capabilities [12].

The Internet is one of the most important technologies used in almost all dimensions of human life, particularly education. This technology is also utilized for distance learning, particularly online education that is one of the prominent virtual education methods [13]. Considering the closure of educational institutions during the COVID-19 pandemic, online education is expanding vastly [14]. This method is used either synchronously or asynchronously [15]. In synchronous online classes, instructors carry out their teaching-learning activities using such software programs as Zoom, Adobe Connect, Minerva, and Blackboard in real time [16]. It should also be noted that learners have freedom in action, speed, and path of learning in electronic learning environments, which can enhance their motivation [17]. According to the results of a study, online classes exerted positive impacts on learners’ academic motivation [18].

Flipped classroom is yet another new teaching methodology resulted from progresses in the field of education. This method is a combination of traditional and digital performances [19]. In this method, out-of-class activities like assignments are transferred into the class and the activities carried out in the class are transferred out of the class [20]. In other words, flipped classroom encourages individuals to learn the educational content at home and practice at school [21]. Some researchers have disclosed that this method enhanced students’ responsibility, sense of belonging to a group, interactions, and motivation [22]. Some studies also revealed that flipped classrooms had a positive effect on students’ academic motivation [23]. However, other studies demonstrated that flipped classrooms had no effects on academic motivation [24].

Nowadays, working in teams and networks is important for solving complicated problems. Therefore, collaborative learning methods have attracted a lot of attention [25]. Jigsaw is one of the collaborative learning methods, in which learners take the responsibility for a part of a task in small predefined groups. Each group member studies the intended issue and then, the members of various teams who have had the responsibility for a similar part create a special group and discuss the intended issue. After that, each member goes back to their groups and teaches the other members [26]. Since learners make attempts and interact for a shared goal, their motivation increases in comparison to competition-based settings [27]. Rachmah considered the jigsaw method as an effective way to improve students’ academic motivation [28]. Nonetheless, another study showed no improvements in the students’ academic motivation by using the jigsaw method [29].

As mentioned earlier, the COVID-19 pandemic has caused numerous challenges in teaching-learning affairs. In this context, instructors try to find methods to maintain and improve students’ motivation, thereby promoting their academic performance. Based on the studies conducted on the issue, online learning [30, 31] and flipped classroom [32, 33] are amongst the most widely-used virtual education methods. Considering the advantages and disadvantages of each of these methods, the combination of flipped classroom and jigsaw method was investigated as a new method in the present study. This study aimed to explore the effects of synchronous online classes and the combination of flipped and jigsaw methods on students’ academic motivation.

## Methods

### Study design and setting

This quasi-experimental study was conducted on two groups of BSc nursing students in School of Nursing and Midwifery affiliated to Shiraz University of Medical Sciences. In this study, two online class methods were compared in terms of their impacts on students’ academic motivation.

### Participants

The BSc nursing students who had entered Shiraz University of Medical Sciences in September and January 2019–2020 and had passed the nursing concepts course were selected through census. The inclusion criteria of the study were being a BSc student at the second semester of nursing at Shiraz University of Medical Sciences and being willing to take part in the research. The exclusion criteria of the study were not completing the questionnaires and being absent for more than one session.

### Sample size

All students who had entered the university in September and January 2019–2020 were enrolled into the study. Totally, 48 students had entered the university in September, 42 ones of whom were willing to cooperate. On the other hand, 55 students had entered the university in January, 47 ones of whom were willing to participate in the research.

### Interventions

This study was conducted online with the cooperation of the virtual college of Shiraz University of Medical Sciences. In order to allocate the classes into two educational methods, the names of the two classes were written on two pieces of paper and they were put in a bag. It was decided that the first piece of paper taken out of the bag would be allocated to the synchronous online class. A person who had no role in the research brought one of the pieces of paper out and, accordingly, the students entering the university in September 2020 were enrolled into the synchronous online class and those entering the university in January were allocated to the combined methods group.

**Fig. 1:**
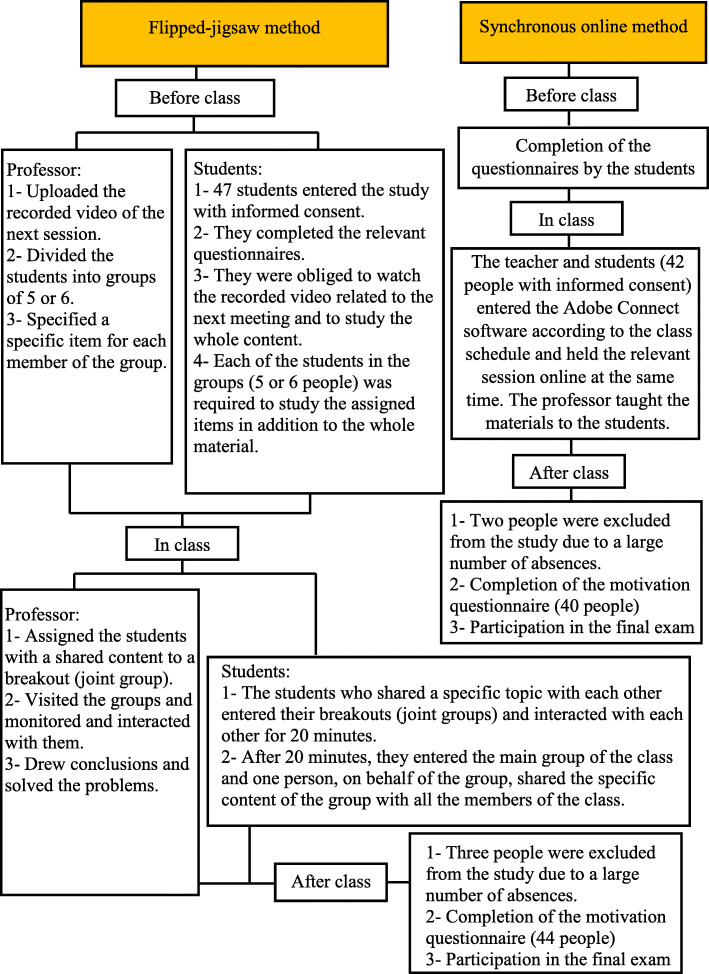
Flow diagram of the interventions

Prior to the intervention, the students in the synchronous online class were provided with some information regarding the research procedures and their electronic informed consent forms were obtained. Out of the 48 students in this group, 42 were willing to take part in the study. Demographic and academic motivation questionnaires were electronically sent to the students before the intervention. It should be noted that the students who did not take part in the study passed the course offline. The basic concepts of nursing were taught using Adobe Connect software through nine sessions in synchronous online classes. In doing so, the instructor (corresponding author) shared the related slides in the software and taught the materials online. It should be mentioned that two students were excluded from this group because of repeated absences. After the end of the nine education sessions, the 40 students were required to complete the Academic Motivation Scale again (Fig. 1).

In the flipped-jigsaw group, the students were provided with some explanations about the study procedure and their electronic informed consent forms were obtained. Out of the 55 students, 47 were willing to cooperate. The students who were not willing to take part in the research passed the course offline. Before the intervention, demographic and academic motivation questionnaires were sent to the students electronically. In this group, instruction was carried out using a combination of flipped and jigsaw methods for nine sessions. In doing so, a video clip of the intended subject together with some questions were uploaded on the E-learning management system by the instructor (corresponding author) a week prior to each education session. Thus, the students could prepare themselves by watching the video clip or reviewing other resources such as the electronic book by Brunner and Suddarth, 2018 [34] during the week. In each education session, the students were divided into groups of five or six. Each member of the group was assigned a specific topic. They were required to study the whole material, with a closer look at the assigned topic. During the online classes, the students who had similar assigned items entered their joint groups using ‘breakout’ in Adobe Connect software and discussed the points for 20 min. Meanwhile, the instructor monitored the students and answered their questions. After 20 min, the students entered the main group of the class. Then, in order to improve the online work process, one of the students on behalf of their joint groups provided the class with the final summary of the material. In the end, the instructor answered the students’ questions and provided them with a summary. It should be noted that three students were excluded from this group because of repeated absences. After the end of the nine sessions, the Academic Motivation Scale was completed by the 44 students (Fig. 1). It should also be noted that the students in both groups were followed up using WhatsApp. In addition, calling the roll was done electronically using Adobe Connect software and the students’ activities were recorded. Furthermore, the students were able to ask the instructor their questions or problems through the E-learning management system or WhatsApp.

### Ethical consideration

The present study was approved by the Ethics Committee of Shiraz University of Medical Sciences (IR.SUMS.REC.1399.454). All necessary permissions for conducting the research were obtained from the relevant authorities and all methods were performed in accordance with the relevant guidelines and regulations. Furthermore, a session was held after the selection of the participants for explaining the study objectives and procedures. Online informed consent forms were also taken from all the participants.

### Instrument

The study data were collected using a demographic information form and Harter’s Academic Motivation Scale. The demographic information form included age, sex, and Grade Point Average (GPA).

Academic motivation was evaluated using Harter’s Academic Motivation Scale, which was designed in 1981. This questionnaire measures academic motivation using dichotomous questions, one of whose options involves intrinsic motivation and the other involves extrinsic motivation. Harter noted that “situations can be imagined in which internal interest and external rewards, in cooperation with each other, stimulate learning” [35, 36]. This questionnaire was modified by Leper et al. in 2005. In the modified questionnaire, separate questions were designed for intrinsic and extrinsic motivations. This form consisted of 33 items assessing intrinsic (*n* = 17) and extrinsic (*n* = 16) motivations [36, 37]. Intrinsic motivation refers to the satisfaction with and joy of learning (e.g., ‘I ask questions in the class, because I want to learn new things’). Extrinsic motivation, on the other hand, involves achieving positive outcomes and avoiding negative learning outcomes (e.g., ‘When I make a mistake, I like to ask the teacher how to find the right answer’ [38]. In other words, the activities individuals do for themselves are called intrinsic motivation, while the motivation derived from external outcomes is considered extrinsic motivation [39]. The questionnaire items could be responded based on a Likert scale (never = 1, rarely = 2, sometimes = 3, usually = 4, and almost always = 5). Thus, the total score of the questionnaire could range from 33 to 165 [12]. Lepper et al. confirmed the predictive validity of the modified scale through the significant correlation between intrinsic motivation and the instructor’s reports of intrinsic motivation. Significant correlations were also observed between intrinsic and extrinsic motivation and their subscales and two objective indices of academic progress; i.e., course scores and academic progress scores. Additionally, the test-retest coefficient was found to be 0.74 for both intrinsic and extrinsic motivations [37]. In Harter’s study, the reliability coefficients of the two subscales ranged from 0.54 to 0.84 using the Kuder-Richardson 20 formula. In addition, the reliability coefficients ranged from 0.48 to 0.63 in a sample during a nine-month period and from 0.58 to 0.76 in another sample during 5 months [35]. In Iran, the reliability and validity of this scale were approved by Behroozi. In that study, the validity of the scale was confirmed by confirmatory factor analysis. Additionally, its reliability was confirmed by Cronbach’s alpha coefficient of 0.72 for intrinsic motivation and 0.71 for extrinsic motivation [40]. In another study also, the reliability of the scale was confirmed by Cronbach’s alpha = 0.79 [12].

### Statistical analysis

The normality of the data was assessed using the Kolmogorov-Smirnov test. Accordingly, all demographic data and other study variables followed normal distribution. Descriptive statistics were used for the demographic data and the variables were reported as mean, standard deviation, frequency, and percentage. Independent t-test and chi-square test were applied for comparing the two groups regarding the demographic variables. Moreover, the pre- and post-intervention scores were compared in the two groups via paired t-test. Finally, ANCOVA was used to compare the differences between the two groups’ scores.

## Results

### Participants’ characteristics

This study was conducted on 40 students in the synchronous online class and 44 ones in the flipped-jigsaw group. As Table 1 shows, there were more female students in both groups (52.5% in the synchronous online class and 52.3% in the flipped-jigsaw group). In the synchronous online group, the mean age of the students was 20.88 + 3.164 years and their mean GPA was 14.595 + 1.929. In the flipped-jigsaw group, the mean age of the students was 21.27 + 3.128 years and their mean GPA was 16.980 + 0.744. The results indicated that the groups were homogeneous with regard to age and sex, but were significantly different regarding GPA (*p* < 0.001) (Table 1). As the GPA was different between the two groups, ANCOVA was used for between-group comparisons.

**Table.1:** Demographic data of the participants

		Synchronous online class	Flipped-jigsaw learning	*P*-value
Sex n (%)	Female	21 (52.5%)	23 (52.3%)	0.983
	Male	19 (47.5%)	21 (47.7%)	
GPA (M ± SD)		14.595 ± 1.929	16.980 ± 0.744	< 0.001
Age (M ± SD)		20.88 ± 3.164	21.27 ± 3.128	0.564

### Academic motivation

The results showed no significant difference between the two groups concerning the mean scores of academic motivation and its intrinsic and extrinsic dimensions before the intervention (Table 2). In the synchronous online group, no significant increase was observed in the intrinsic and extrinsic dimensions of academic motivation after the intervention. However, a significant increase was found in the mean scores of academic motivation (*p* = 0.002) and its intrinsic (*p* = 0.003) and extrinsic (*p* = 0.031) dimensions in the flipped-jigsaw group after the intervention. Furthermore, the results of ANCOVA indicated that the mean scores of academic motivation (*p* = 0.007) and its intrinsic (*p* = 0.038) and extrinsic (*p* = 0.010) dimensions were significantly higher in the flipped-jigsaw group compared to the synchronous online class after the intervention.

**Table.2:** Comparison of the two groups regarding the mean scores of academic motivation and its dimensions

Variable	Groups	Before (Mean ± SD)	After (Mean ± SD)	*P*-value (within-group)^a^
Intrinsic motivation	Synchronous online	58.125 ± 7.490	58.500 ± 8.958	0.725
	Flipped-jigsaw	60.772 ± 8.569	65.045 ± 7.351	0.003
	P-value (between-group)^b^	0.461	0.038	
Extrinsic motivation	Synchronous online	48.575 ± 5.597	49.850 ± 5.681	0.131
	Flipped-jigsaw	50.727 ± 7.289	53.840 ± 7.736	0.031
	P-value (between-group) ^b^	0.226	0.010	
Academic motivation (Total)	Synchronous online	106.700 ± 11.223	108.350 ± 12.263	0.229
	Flipped-jigsaw	111.500 ± 14.342	118.886 ± 13.026	0.002
	P-value (between-group) ^b^	0.285	0.007	

^a^ Paired t-test for within-group comparisons

^b^ ANCOVA for between-group comparisons

## Discussion

This study aimed to compare the effects of two common educational methods on students’ academic motivation during the COVID-19 pandemic. The results demonstrated that the combination of flipped and jigsaw methods had a more positive effect on the students’ academic motivation and its dimensions compared to the synchronous online class.

The findings indicated that the two groups were homogeneous regarding sex and age. However, the mean GPA was higher in the flipped-jigsaw group compared to the synchronous online class. This might be due to the fact that the participants were selected from the students who had entered the university in different years. In case the participants had been selected from a group of students entering the university in the same year, they would have had the opportunity to interact with each other, which could eventually affect the study results. Moreover, in this case, the students had to be divided into two very small groups. Another reason for the difference in GPA was that all the students had to pass this course in the second semester, which could make a difference in the two groups’ GPAs over time.

The results revealed a significant increase in the mean scores of academic motivation and its intrinsic and extrinsic dimensions in the flipped-jigsaw group after the intervention. However, this was not the case in the synchronous online class. Furthermore, a significant change was observed in the students’ academic motivation in the flipped-jigsaw group compared to the synchronous online class after the intervention. Mohammad stated that motivation played an important role in learning. Accordingly, lack of motivation would be accompanied by no learning activity [41]. Hence, motivation could be considered an important factor in raising the level of success [42]. The results of a previous study indicated that the increase in the number of online sessions, lack of facilities (such as adequate Internet networks), and cost of online classes were effective factors in reducing academic motivation amongst students [43]. In the present study, the students in the synchronous online class only listened to the educational content at the same time, and the teacher-centeredness of the training sessions reduced the students’ interactions with each other as well as with the teacher. These conditions eventually decreased the students’ motivation in the synchronous online class. In contrast, Dliss and Sukur reported that the students who were able to use online classes during the COVID-19 pandemic were more motivated compared to those who could not benefit from online education [44]. The findings of a prior study demonstrated that due to students’ readiness before beginning of the class, division of tasks that takes place at home and in the classroom, and attractiveness of the method, the flipped classroom method was effective in improving students’ academic motivation [45]. Allocating time to interactive activities, increasing learners’ interest and participation, giving students the responsibility for learning, and having access to educational materials at any time of the day can also be mentioned as other factors in promoting academic motivation [46]. In another study, the Jigsaw teaching method was found to be effective in increasing nursing students’ academic motivation due to being challenging and providing the ground for discussion among students [41]. In the present study, the flipped-jigsaw method was effective in increasing academic motivation due to being student-centered, helping the students be sufficiently prepared before entering the classroom that gave them a feeling of security, giving responsibility to the students, placing students in small groups that enhanced their interaction with each other as well as with the teacher, and being attractive and challenging. Generally, existence of competition for high grades, assignments, and student-centered activities can be effective in promoting students’ intrinsic and extrinsic motivation [47]. In the present study, the flipped-jigsaw method was more effective in promoting the aforementioned points in comparison to the synchronous online class, which ultimately led to the improvement of intrinsic and extrinsic motivation scores amongst the students. A previous research revealed that intrinsic motivation was more effective in academic achievement compared to extrinsic motivation [47]. The present study findings showed that the combination of flipped and jigsaw methods promoted motivation in the students. Accordingly, a significant increase was observed in both intrinsic and extrinsic dimensions among the students in the flipped-jigsaw class, but not among those in the synchronous online class. Therefore, this combined method that was effective in improving the students’ motivation was suitable for their academic progress.

One of the limitations of this study was that it could not be compared to the lecture method. Hence, future studies are recommended to compare the flipped-jigsaw method to the lecture method. Furthermore, the participants were selected from the students entering the university in different years so as to avoid interaction among the participants. Thus, further investigations are suggested to be performed on a large number of students entering a university in 1 year. However, one of the strengths of this study was that the two groups were in exactly the same situation, so that only education methods could make a difference.

## Conclusion

Considering the COVID-19 pandemic, educational institutions have had to make use of virtual education methods. This has resulted in a change in instructors’ teaching methods, which can be effective in students’ process of education. In this context, instructors make attempts to find methods for maintaining and improving students’ motivation, eventually promoting their academic performance. The findings of the present study demonstrated that the combination of flipped and jigsaw methods was more effective in improvement of the students’ academic motivation in comparison to the synchronous online class.

## Data Availability

The datasets used and/or analyzed during the current study are available from the corresponding author on reasonable request.

## Abbreviations

BSc: Bachelor of Science
E-learning: Electronic learning
GPA: Grade Point Average
M ± SD: Mean ± Standard Deviation
